# 6,8-Di­chloro-3-(pyridin-2-yl)-2-[1-(pyridin-2-yl)eth­yl]-1,2-di­hydro­quinoxaline

**DOI:** 10.1107/S241431462300665X

**Published:** 2023-08-04

**Authors:** Frederick P. Malan, Ahmed M. Mansour, Amanda-Lee E. Manicum

**Affiliations:** a Department of Chemistry, University of Pretoria, 0002, Pretoria, South Africa; b Department of Chemistry, Faculty of Science, Cairo University, Gamma Street, Giza, Cairo 12613, Egypt; c Department of Chemistry, Tshwane, University of Technology, 0001, Pretoria, South Africa; University of Aberdeen, United Kingdom

**Keywords:** crystal structure, quinoxaline derivative, chiral compound

## Abstract

The crystal structure of the racemic title compound, C_20_H_16_C_l2_N_4_ is described, where the formation of a di-substituted 6,8-di­chloro quinoxaline, containing two stereogenic centres, is confirmed.

## Abstract 2

The synthesis and single-crystal X-ray structure of a substituted quinoxaline compound is described.

## Graphical abstract

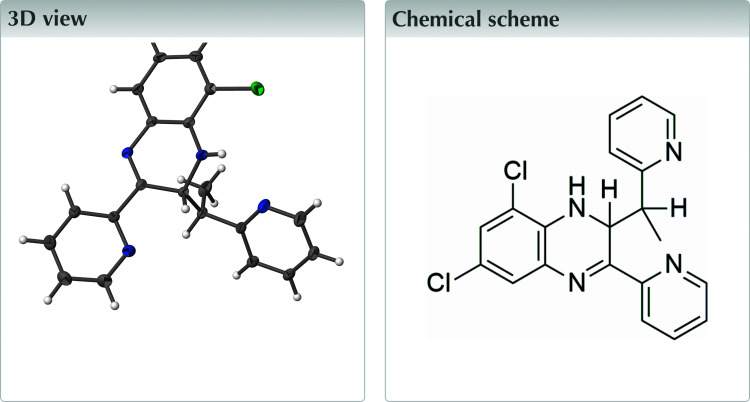

## Structure description

The family of functionalized quinoxaline compounds is an important class of heterocyclic compounds because of their synthetic utility and electroluminescent properties, as well as the different biological properties they have been found to exhibit (Pereira *et al.*, 2015 ▸). The gradually expanding library of active compounds has lead to a growing inter­est into their solid- and solution-state characterization, including single-crystal X-ray diffraction. As part of our studies in this area, we now describe the synthesis and structure of the title compound, C_20_H_16_C_l2_N_4_.

**Figure 1:**
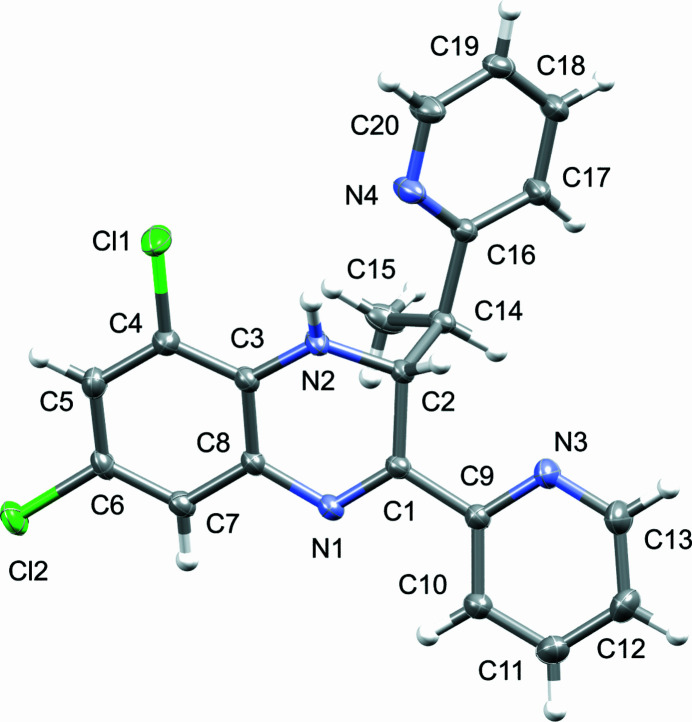
Perspective view of the mol­ecular structure of the title compound showing displacement ellipsoids at the 50% probability level.

The compound crystallizes in the monoclinic space group *P*2_1_/*c* with *Z* = 4. The asymmetric unit (Fig. 1 ▸) contains one mol­ecule, featuring the 6,8-di­chloro­quinoxaline-based skeleton with two pyridyl-based substituents attached to positions 2 and 3 (atoms C1 and C2, respectively). The compound contains two chiral centres, namely atoms C3 and C14: in the arbitrarily chosen asymmetric unit, these both have an *R* configuration, but crystal symmetry generates a racemic mixture. The quinoxalinyl ring system and the 2-pyridyl groups are close to co-planar [N3—C9—C1—N1 = −179.61 (14), C8—N1—C1—C9 = 175.17 (13)°], with the third picolyl-containing substituent more notably rotated out of plane [C1—C2—C14—C16 = −166.73 (12)°] with respect to the quinoxalinyl group. In the quinoxaline moiety, partial saturation on C2 (position 3) occurs and C2 is *sp* ^3^-hybridized with bond angles of 113.58 (12)° (N2—C2—C14), 108.58 (12)° (N2—C2—C1) and 112.34 (12)° (C1—C2—C14). This leads C2 to be displaced by 0.383 (3) Å from the quinoxalinyl mean plane. Bonds lengths supporting the partially saturated character include: 1.290 (2) Å (N1—C1), 1.522 (2) Å (C1—C2), 1.4586 (19) Å (C2—N2) and 1.550 (2) Å (C2—C14). The remaining C—C, C—Cl, and C—N bond lengths and angles agree well with similar pyridyl-containing quinoxaline systems (Wang *et al.*, 2015 ▸). A weak bifurcated intra­molecular N—H⋯(N,Cl) hydrogen bond occurs (Table 1 ▸).

**Figure 2:**
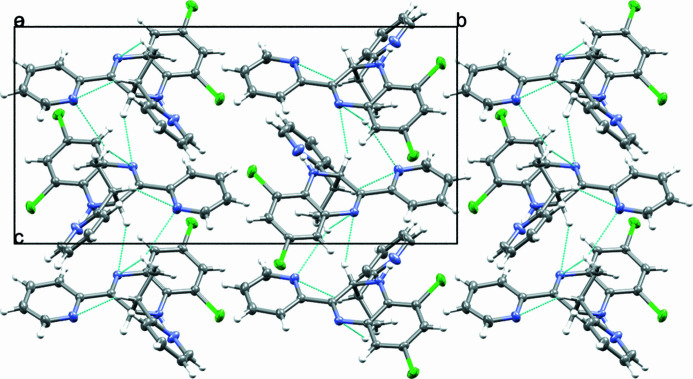
Packing viewed along the *a*-axis direction. Hydrogen-bonding inter­actions are indicated by means of cyan lines.

In the crystal, the compound packs as layers that extend down the *c*-axis inter­linked by weak C—H⋯N hydrogen-bonding inter­actions (Fig. 2 ▸). No aromatic π–π stacking inter­actions were observed.

## Synthesis and crystallization

Picolyl­amine (1 mmol), 2-methyl-2-(2-pyrid­yl)ethyl­amine (1 mmol) and 3,5-di­chloro­cyclo­hexan-1,2-dione (1 mmol) were added to a round-bottom flask with methanol (20 ml). The resulting solution was carefully heated to 50°C for approximately 2 h. The yellow solution was left to crystallize, after which yellow crystals of the title compound (which in this case represents the major product) were obtained.

## Refinement

Crystal data, data collection and structure refinement details are summarized in Table 2 ▸. The highest calculated residual electron density is 0.62 e Å^−3^ at 0.91 Å from N2.

## Supplementary Material

Crystal structure: contains datablock(s) I. DOI: 10.1107/S241431462300665X/hb4436sup1.cif

Structure factors: contains datablock(s) I. DOI: 10.1107/S241431462300665X/hb4436Isup2.hkl

Supporting information file. DOI: 10.1107/S241431462300665X/hb4436Isup3.cdx

Supporting information file. DOI: 10.1107/S241431462300665X/hb4436Isup4.cml

CCDC reference: 2285764

Additional supporting information:  crystallographic information; 3D view; checkCIF report

## Figures and Tables

**Table 1:** Hydrogen-bond geometry (Å, °)

*D*—H⋯*A*	*D*—H	H⋯*A*	*D*⋯*A*	*D*—H⋯*A*
N2—H2⋯Cl1	0.88	2.64	2.9941 (13)	105
N2—H2⋯N4	0.88	2.50	2.815 (2)	102

**Table 2:** Experimental details

Crystal data	
Chemical formula	C_20_H_16_Cl_2_N_4_
*M*_r_	383.27
Crystal system, space group	Monoclinic, *P*2_1_/*c*
Temperature (K)	150
*a*, *b*, *c* (Å)	8.4245 (3), 20.7040 (6), 10.2055 (3)
β (°)	96.448 (3)
*V* (Å^3^)	1768.79 (10)
*Z*	4
Radiation type	Mo *K*α
μ (mm^−1^)	0.38
Crystal size (mm)	0.27 × 0.19 × 0.09

Data collection	
Diffractometer	XtaLAB Synergy R, DW system, HyPix
Absorption correction	Multi-scan (*CrysAlis PRO*; Rigaku OD, 2019)
*T*_min_, *T*_max_	0.576, 1.000
No. of measured, independent and observed [*I* > 2σ(*I*)] reflections	29036, 4742, 3981
*R*_int_	0.112
(sin θ/λ)_max_ (Å^−1^)	0.719

Refinement	
*R*[*F* ^2^ > 2σ(*F* ^2^)], *wR*(*F* ^2^), *S*	0.047, 0.137, 1.10
No. of reflections	4742
No. of parameters	236
H-atom treatment	H-atom parameters constrained
Δρ_max_, Δρ_min_ (e Å^−3^)	0.62, −0.58

Computer programs: *CrysAlis PRO* (Rigaku OD, 2023 ▸), *SHELXT* (Sheldrick, 2015*a* ▸), *SHELXL* (Sheldrick, 2015*b* ▸) and *OLEX2* (Dolomanov *et al.*, 2009 ▸).

## full crystallographic data

### Crystal data

**Table:**

C_20_H_16_Cl_2_N_4_	*F*(000) = 792
*M**_r_* = 383.27	*D*_x_ = 1.439 Mg m^−^^3^
Monoclinic, *P*2_1_/*c*	Mo *K*α radiation, λ = 0.71073 Å
*a* = 8.4245 (3) Å	Cell parameters from 18589 reflections
*b* = 20.7040 (6) Å	θ = 2.6–31.0°
*c* = 10.2055 (3) Å	µ = 0.38 mm^−^^1^
β = 96.448 (3)°	*T* = 150 K
*V* = 1768.79 (10) Å^3^	Blade, yellow
*Z* = 4	0.27 × 0.19 × 0.09 mm

### Data collection

**Table:**

XtaLAB Synergy R, DW system, HyPix diffractometer	4742 independent reflections
Radiation source: Rotating-anode X-ray tube, Rigaku (Mo) X-ray Source	3981 reflections with *I* > 2σ(*I*)
Mirror monochromator	*R*_int_ = 0.112
Detector resolution: 10.0000 pixels mm^-1^	θ_max_ = 30.8°, θ_min_ = 2.4°
ω scans	*h* = −12→10
Absorption correction: multi-scan (CrysalisPro; Rigaku OD, 2019)	*k* = −28→26
*T*_min_ = 0.576, *T*_max_ = 1.000	*l* = −14→13
29036 measured reflections	

### Refinement

**Table:**

Refinement on *F*^2^	Primary atom site location: dual
Least-squares matrix: full	Hydrogen site location: inferred from neighbouring sites
*R*[*F*^2^ > 2σ(*F*^2^)] = 0.047	H-atom parameters constrained
*wR*(*F*^2^) = 0.137	*w* = 1/[σ^2^(*F*_o_^2^) + (0.0676*P*)^2^ + 0.7299*P*] where *P* = (*F*_o_^2^ + 2*F*_c_^2^)/3
*S* = 1.10	(Δ/σ)_max_ = 0.001
4742 reflections	Δρ_max_ = 0.62 e Å^−^^3^
236 parameters	Δρ_min_ = −0.58 e Å^−^^3^
0 restraints	

### Special details

**Table:**

Geometry. All esds (except the esd in the dihedral angle between two l.s. planes) are estimated using the full covariance matrix. The cell esds are taken into account individually in the estimation of esds in distances, angles and torsion angles; correlations between esds in cell parameters are only used when they are defined by crystal symmetry. An approximate (isotropic) treatment of cell esds is used for estimating esds involving l.s. planes.
Refinement. All H atoms were placed in geometrically idealized positions and constrained to ride on their parent atoms.

### Fractional atomic coordinates and isotropic or equivalent isotropic displacement parameters (Å^2^)

**Table:**

	*x*	*y*	*z*	*U*_iso_*/*U*_eq_	
Cl2	1.12383 (5)	0.09926 (2)	0.40380 (4)	0.02882 (13)	
Cl1	0.83348 (6)	0.03634 (2)	0.83287 (5)	0.03650 (15)	
N1	0.81871 (15)	0.26674 (6)	0.64452 (13)	0.0189 (3)	
N2	0.73638 (16)	0.17552 (6)	0.82624 (13)	0.0201 (3)	
H2	0.7411	0.1527	0.8994	0.024*	
N3	0.59274 (18)	0.36766 (7)	0.83720 (15)	0.0261 (3)	
N4	0.45284 (17)	0.14108 (7)	0.93078 (16)	0.0279 (3)	
C16	0.37494 (18)	0.18777 (7)	0.85957 (15)	0.0179 (3)	
C8	0.85917 (17)	0.20116 (7)	0.63224 (15)	0.0181 (3)	
C3	0.81853 (18)	0.15560 (7)	0.72517 (15)	0.0187 (3)	
C9	0.69244 (18)	0.35242 (7)	0.74782 (15)	0.0189 (3)	
C1	0.72267 (17)	0.28236 (7)	0.72928 (15)	0.0175 (3)	
C2	0.64108 (17)	0.23446 (7)	0.81296 (14)	0.0169 (3)	
H2A	0.6423	0.2536	0.9030	0.020*	
C6	1.00485 (19)	0.12035 (8)	0.52627 (16)	0.0215 (3)	
C14	0.46394 (18)	0.22276 (7)	0.75909 (15)	0.0190 (3)	
H14	0.4123	0.2659	0.7422	0.023*	
C7	0.95056 (18)	0.18329 (8)	0.53247 (15)	0.0205 (3)	
H7	0.9755	0.2141	0.4690	0.025*	
C10	0.76783 (19)	0.39916 (8)	0.67700 (17)	0.0224 (3)	
H10	0.8372	0.3868	0.6142	0.027*	
C5	0.96985 (19)	0.07447 (8)	0.61717 (16)	0.0236 (3)	
H5	1.0082	0.0315	0.6126	0.028*	
C4	0.8772 (2)	0.09267 (8)	0.71550 (17)	0.0230 (3)	
C17	0.2167 (2)	0.20287 (8)	0.87252 (19)	0.0271 (4)	
H17	0.1635	0.2360	0.8203	0.033*	
C19	0.2179 (2)	0.12117 (8)	1.03820 (18)	0.0263 (3)	
H19	0.1672	0.0977	1.1018	0.032*	
C20	0.3742 (2)	0.10886 (9)	1.01806 (19)	0.0300 (4)	
H20	0.4295	0.0757	1.0688	0.036*	
C11	0.7391 (2)	0.46377 (8)	0.70050 (19)	0.0288 (4)	
H11	0.7886	0.4965	0.6541	0.035*	
C12	0.6367 (2)	0.47994 (9)	0.7931 (2)	0.0325 (4)	
H12	0.6154	0.5238	0.8118	0.039*	
C13	0.5664 (2)	0.43017 (9)	0.8576 (2)	0.0330 (4)	
H13	0.4954	0.4414	0.9199	0.040*	
C18	0.1374 (2)	0.16896 (9)	0.9625 (2)	0.0305 (4)	
H18	0.0290	0.1784	0.9722	0.037*	
C15	0.4450 (2)	0.18517 (10)	0.62926 (16)	0.0290 (4)	
H15A	0.4889	0.1416	0.6441	0.044*	
H15B	0.5026	0.2076	0.5644	0.044*	
H15C	0.3315	0.1822	0.5961	0.044*	

### Atomic displacement parameters (Å^2^)

**Table:**

	*U*^11^	*U*^22^	*U*^33^	*U*^12^	*U*^13^	*U*^23^
Cl2	0.0285 (2)	0.0321 (2)	0.0284 (2)	0.00069 (15)	0.01460 (17)	−0.00866 (15)
Cl1	0.0531 (3)	0.0222 (2)	0.0386 (3)	0.00890 (18)	0.0247 (2)	0.00922 (17)
N1	0.0183 (6)	0.0205 (6)	0.0189 (6)	0.0000 (5)	0.0063 (5)	0.0003 (5)
N2	0.0214 (6)	0.0216 (6)	0.0188 (6)	0.0054 (5)	0.0087 (5)	0.0045 (5)
N3	0.0305 (7)	0.0215 (7)	0.0287 (7)	0.0010 (5)	0.0135 (6)	−0.0030 (5)
N4	0.0194 (7)	0.0333 (8)	0.0324 (8)	0.0030 (6)	0.0085 (6)	0.0137 (6)
C16	0.0175 (7)	0.0186 (7)	0.0182 (7)	−0.0023 (5)	0.0043 (5)	0.0000 (5)
C8	0.0163 (7)	0.0203 (7)	0.0184 (7)	0.0002 (5)	0.0054 (5)	−0.0002 (5)
C3	0.0170 (7)	0.0207 (7)	0.0192 (7)	0.0010 (5)	0.0054 (6)	0.0005 (5)
C9	0.0180 (7)	0.0188 (7)	0.0205 (7)	−0.0004 (5)	0.0044 (6)	−0.0006 (5)
C1	0.0159 (7)	0.0192 (7)	0.0179 (7)	−0.0007 (5)	0.0040 (5)	0.0012 (5)
C2	0.0168 (7)	0.0176 (7)	0.0172 (7)	0.0008 (5)	0.0056 (5)	0.0006 (5)
C6	0.0195 (7)	0.0256 (8)	0.0206 (7)	0.0008 (6)	0.0074 (6)	−0.0066 (6)
C14	0.0163 (7)	0.0217 (7)	0.0196 (7)	0.0002 (5)	0.0047 (5)	0.0053 (6)
C7	0.0209 (7)	0.0226 (8)	0.0190 (7)	−0.0017 (6)	0.0071 (6)	−0.0016 (6)
C10	0.0205 (7)	0.0220 (8)	0.0254 (8)	−0.0009 (6)	0.0062 (6)	0.0017 (6)
C5	0.0241 (8)	0.0208 (8)	0.0266 (8)	0.0031 (6)	0.0059 (6)	−0.0035 (6)
C4	0.0249 (8)	0.0204 (7)	0.0248 (8)	0.0014 (6)	0.0085 (6)	0.0018 (6)
C17	0.0213 (8)	0.0252 (8)	0.0368 (9)	0.0047 (6)	0.0116 (7)	0.0079 (7)
C19	0.0277 (8)	0.0256 (8)	0.0276 (8)	−0.0060 (6)	0.0120 (7)	0.0010 (6)
C20	0.0239 (8)	0.0332 (9)	0.0337 (9)	0.0010 (7)	0.0068 (7)	0.0146 (7)
C11	0.0287 (9)	0.0221 (8)	0.0361 (9)	−0.0044 (6)	0.0056 (7)	0.0010 (7)
C12	0.0383 (10)	0.0196 (8)	0.0407 (10)	0.0001 (7)	0.0093 (8)	−0.0041 (7)
C13	0.0409 (10)	0.0247 (9)	0.0363 (10)	0.0023 (7)	0.0177 (8)	−0.0053 (7)
C18	0.0231 (8)	0.0275 (9)	0.0442 (10)	0.0020 (6)	0.0176 (7)	0.0059 (7)
C15	0.0264 (8)	0.0428 (10)	0.0181 (7)	−0.0098 (7)	0.0033 (6)	−0.0003 (7)

### Geometric parameters (Å, º)

**Table:**

Cl2—C6	1.7432 (16)	C9—C1	1.488 (2)
Cl1—C4	1.7406 (17)	C9—C10	1.402 (2)
N1—C8	1.409 (2)	C1—C2	1.522 (2)
N1—C1	1.290 (2)	C2—C14	1.550 (2)
N2—C3	1.3687 (19)	C6—C7	1.385 (2)
N2—C2	1.4586 (19)	C6—C5	1.382 (2)
N3—C9	1.345 (2)	C14—C15	1.529 (2)
N3—C13	1.333 (2)	C10—C11	1.385 (2)
N4—C16	1.336 (2)	C5—C4	1.391 (2)
N4—C20	1.346 (2)	C17—C18	1.385 (2)
C16—C14	1.520 (2)	C19—C20	1.379 (2)
C16—C17	1.390 (2)	C19—C18	1.385 (3)
C8—C3	1.407 (2)	C11—C12	1.390 (3)
C8—C7	1.394 (2)	C12—C13	1.391 (3)
C3—C4	1.401 (2)		
			
C1—N1—C8	118.51 (13)	C1—C2—C14	112.34 (12)
C3—N2—C2	120.11 (13)	C7—C6—Cl2	119.20 (13)
C13—N3—C9	117.45 (15)	C5—C6—Cl2	119.49 (12)
C16—N4—C20	118.06 (14)	C5—C6—C7	121.29 (14)
N4—C16—C14	117.53 (13)	C16—C14—C2	111.30 (12)
N4—C16—C17	121.91 (15)	C16—C14—C15	109.31 (13)
C17—C16—C14	120.53 (14)	C15—C14—C2	112.90 (13)
C3—C8—N1	120.43 (13)	C6—C7—C8	119.71 (15)
C7—C8—N1	118.68 (14)	C11—C10—C9	118.64 (16)
C7—C8—C3	120.70 (14)	C6—C5—C4	118.51 (15)
N2—C3—C8	119.20 (14)	C3—C4—Cl1	118.10 (12)
N2—C3—C4	123.06 (14)	C5—C4—Cl1	119.63 (12)
C4—C3—C8	117.49 (14)	C5—C4—C3	122.26 (15)
N3—C9—C1	116.33 (14)	C18—C17—C16	119.20 (15)
N3—C9—C10	122.77 (15)	C20—C19—C18	117.70 (16)
C10—C9—C1	120.88 (14)	N4—C20—C19	123.81 (16)
N1—C1—C9	117.29 (14)	C10—C11—C12	118.95 (17)
N1—C1—C2	124.72 (13)	C11—C12—C13	118.25 (17)
C9—C1—C2	117.98 (13)	N3—C13—C12	123.93 (17)
N2—C2—C1	108.58 (12)	C19—C18—C17	119.30 (16)
N2—C2—C14	113.58 (12)		

### Hydrogen-bond geometry (Å, º)

**Table:**

*D*—H···*A*	*D*—H	H···*A*	*D*···*A*	*D*—H···*A*
N2—H2···Cl1	0.88	2.64	2.9941 (13)	105
N2—H2···N4	0.88	2.50	2.815 (2)	102
